# Fluid escapes to the “third space” during anesthesia, a commentary

**DOI:** 10.1111/aas.13740

**Published:** 2020-11-24

**Authors:** Robert G. Hahn

**Affiliations:** ^1^ Research Unit Södertälje Hospital Södertälje Sweden; ^2^ Karolinska Institutet at Danderyds Hospital (KIDS) Stockholm Sweden

## Abstract

**Background:**

The “third fluid space” is a concept that has caused much confusion for more than half a century, dividing anesthesiologists into believers and non‐believers.

**Aim:**

To challenge the existence of the “third fluid space” based on analysis of crystalloid fluid kinetics.

**Methods:**

Data on hemodilution patterns from 157 infusion experiments performed in volunteers and from 85 patients undergoing surgery under general anesthesia were studied by population volume kinetic analysis. Elimination of infused crystalloid fluid from the kinetic model could occur either as urine or "third space" accumulation. The latter fluid volume remained in the body, but without equilibrating with the plasma within the 3‐4 h of the experiment.

**Results:**

The rate constant for "third space" loss of fluid accounted for 20% of the elimination in conscious volunteers and for 75% during general anesthesia and surgery. The two elimination constants showed a reciprocal relationship, resulting in that "third‐space" losses increase when urinary excretion is restricted. The effect on the plasma volume was smaller than indicated by these figures because fluid distributed to the extravascular space continuously redistributed to the plasma. Worked‐out examples show that one‐third of an infused crystalloid volume has been confined to the "third space" after 3 h of surgery. When equilibration with the plasma eventually occurs, which is necessary for excretion of the fluid, is not known.

**Conclusion:**

During anesthesia and surgery one third of the infused crystalloid fluid is at least temporarily unavailable for excretion, which probably contributes to postoperative weight increase and edema.

**Editorial Comment**

The “third” space in clinical medicine has been discussed for many decades, lightly described by some as an unreachable compartment of unclear dimension. In this commentary, earlier and current ideas concerning intravascular fluid regress from vessels during the perioperative setting are presented.

## 1 INTRODUCTION

Fluid overload is a frequent consequence of anesthesia and surgery and is statistically associated with postoperative complications.^1^ Infusion of more than 3 L of crystalloid has been linked to prolonged gastrointestinal recovery time,^2^ and 5‐6 L increases the risk of poor wound healing, pulmonary congestion, and pulmonary edema.^3^, ^4^, ^5^, ^6^, ^7^ In abdominal operations, suture insufficiency occurs more easily and this might lead to sepsis.^7^

A recent study (RELIEF) suggests benefits of large crystalloid volumes,^8^ but these results should be interpreted with caution because one‐third of the patients underwent abdominal surgery after enteric lavage, which causes volume depletion.^9^ Massive documentation still shows that liberal fluid administration causes harm.

## 2 WHY WE INFUSE SO MUCH FLUID?

Apart from replacement of hemorrhage and evaporative fluid losses, three factors can explain why we infuse fluid during surgery in amounts that exceed the minimal requirement of 1 mL/kg/h in conscious humans.

1. Anesthesia causes vasodilatation that increases the unstressed blood volume. To maintain venous return, the enlarged "vascular costume" must be filled up with infusion fluid or (more slowly) be replaced by endogenous capillary refill.
2. The disturbance of the autonomous function redistributes blood flows. Just like a high spinal anesthesia, general anesthesia causes a mild distributive shock that needs to be treated with fluid and/or vasoconstrictors. A likely evidence of such redistribution is that infusion of >2 mL/kg/h during surgery is needed to prevent postoperative nausea.^10^
3. Drug‐induced reduction of the arterial pressure is confused with hypovolemic hypotension.

## 3 WHY DOES LONG‐STANDING EDEMA DEVELOP?

Our kidneys do correct overload within a few hours if we ingest too much liquid in the conscious state. A relevant question is then why fluid given during an operation causes an increase in body weight that lasts several days?^7^

Volume kinetic studies performed during the past 20 years show that the capacity to eliminate a crystalloid fluid load during general anesthesia is reduced by 80%‐90% as compared to the conscious state. The key factor for this dramatic reduction appears to be the anesthesia‐induced decrease of the arterial pressure.^11^ As soon as the pressure is restored after the surgery, a normal diuretic response to fluid returns, but the overload developing during the surgery is difficult to eliminate. Why?

Fluid‐retaining hormones and the “stress response to surgery” have long been implicated as the mechanism for the weight increase after surgery. However, this explanation is inconsistent with the rapid return of a normal diuretic response as soon as the patients wakes up,^12^ and the fact that the diuretic response to fluid loading is stronger 4 hours after cholecystectomy than before the operation.^13^ Moreover, restrictive fluid therapy after surgery leads to more complications, not fewer.^14^

One possible mechanism is that fluid loading changes the elastic properties of the interstitial matrix, thereby causing edema by promoting a shift in the fluid equilibrium between the plasma and the interstitium.^15^ However, impaired return of distributed fluid due to changes in interstitial compliance is associated with infusing fluid faster than >50 mL/min,^15^ which is rarely done during surgery.

A second mechanism that could serve to explain why fluid infused during surgery remains for several days in the body could be that fluid accumulates somewhere where it does not easily equilibrate with the plasma. This idea is reminiscent of the concept of the “third fluid space” that was popularized in the 1960s but has since then either been forgotten or refuted. Let us look at this possibility.

## 4 A MURKY CONCEPT

In 1960, Shires *et al* found that hemorrhage is followed by a reduction in the extracellular fluid (ECF) volume beyond what could be expected from the blood loss alone.^16^ A similar reduction also occurred during surgery, regardless of hemorrhage volume.^17^ This contraction was thought to arise due to sequestration of fluid to a non‐anatomic “third fluid space” that does not exchange with the circulating plasma.^18^ The decrease in the size of the ECV was believed to require treatment by infusion of large amounts of crystalloid fluid, which was a view that caught general acclaim and initiated an era of liberal fluid administration that lasted for more than a decade.

The existence of the “third space” has divided anesthesiologists into believers and non‐believers. Brandstrup et al made a systematic review of the literature up to 2006.^18^ They found diverging results from approximately 40 studies that measured the ECF volume with radioactive tracers in animals and humans during surgery and in shock states. This review, as well as a later one by Jacob et al,^19^ concluded that the “third space” does not have solid evidence and should be regarded as fiction.

## 5 VOLUME KINETICS

A method called “fluid volume kinetics” offers a supplementary view on this topic. Here the distribution and elimination of fluid is analyzed as a function over time without the use of radioactive tracers. The theoretical basis is that changes in blood hemoglobin and red blood cell count during volume therapy mirror the blood water concentration; therefore, their inverse values can be used to create a pharmacokinetic profile of the water volume during and after infusion of any fluid.^12^

**FIGURE 1:**
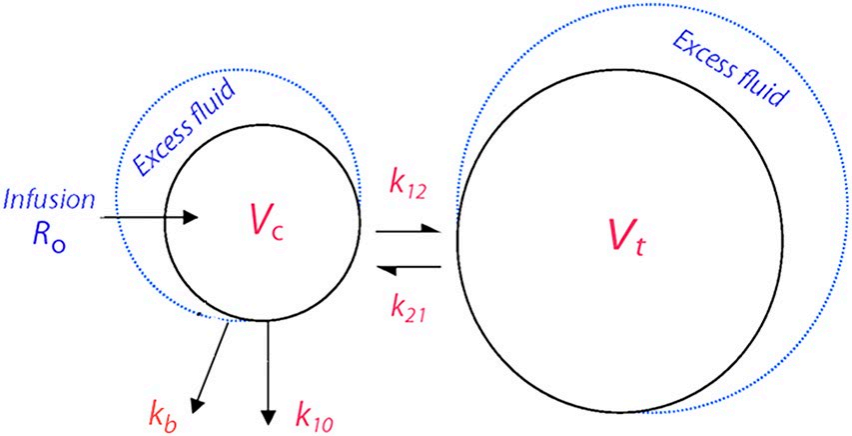
The volume kinetics model, where “third‐spacing” is represented by the rate constant *k*_b_ [Colour figure can be viewed at wileyonlinelibrary.com]

Volume kinetics operates to detect a “wall” between a central (*V*_c_) and a peripheral fluid space (*V*_t_). The exchange of fluid between these spaces is given by two rate constants, *k*_12_ and *k*_21_. The measured urinary excretion equals the rate constant *k*_10_ and “third‐spacing” by the rate constant *k*_b_, which then represents losses of non‐urine fluid from the kinetic system (Figure 1).

The limitations of volume kinetics are mainly restricted to whether the kinetic parameters have physiological correlates. The fluid spaces equilibrating quickly and more slowly with the site of infusion are usually conceived to represent the plasma volume and the expandable part of the interstitial fluid space. The pharmacokinetic parameters of a drug are not usually interpreted in physiological terms due to the complex composite actions of metabolism, transit times, partition coefficients, and osmotic shifts. These considerations do not readily apply to the water volume of isotonic crystalloid fluids.

A recent review argued that much evidence actually supports the idea that volume kinetics reflects the fluid distribution between real body fluid compartments. This evidence includes close correlations between the Hb dilution and isotope measurements of the changes in plasma volume, as well as agreement between the flow pattern generated by *k*_21_ and the lymphatic flow in the thoracic duct.^12^ In two series of volunteer experiments *V*_c_ was recently shown to correlate closely with the baseline plasma volume as obtained by isotope‐validated anthropometric equation^20^

## 6 EARLY FINDINGS OF "THIRD‐SPACING"

In 2002, Brauer et al compared the volume kinetics of 0.9% saline in conscious and isoflurane‐anesthetized sheep.^21^ They found that the kinetic model predicted a far greater elimination of saline from the kinetic system during isoflurane anesthesia, whereas the measured urine volumes showed the opposite effect. A follow‐up study in sheep blamed isoflurane and not the mechanical ventilation for the mismatch, which was then already called “third‐spacing.”^22^ A later study of thyroid surgery patients randomized to receive isoflurane or propofol anesthesia showed no difference in “third‐spacing” between these two modes of anesthesia.^23^ The amount of fluid that “disappeared” was quantified by introducing an elimination term in addition to the urinary excretion in the kinetic model. The “disappeared” fluid averaged 500 mL of an infused volume of 1750 mL during the thyroid surgery, while the fraction was clearly greater in the sheep.^21^, ^22^

Analysis of fluid volume kinetics using the mixed models approach has further expanded our knowledge about this “third‐spacing.” Early insights included that superior curve‐fitting is obtained when *k*_b_ is applied as a first‐order and not a zero‐order constant, which means that the flow of fluid via *k*_b_ increases in proportion to the plasma volume expansion. Better curve‐fitting is also obtained when the “third‐spacing” is assumed to occur from the vascular space and not from the extravascular space.

## 7 “THIRD SPACING” IN VOLUNTEERS AND DURING ANESTHESIA

Approximately 10 articles that cover various clinical settings have been published in which *k*_b_ is reported. A key finding is that *k*_b_ increases when *k*_10_ decreases. To illustrate the proportions, the *k*_b_ value was calculated from a database with 157 infusion experiments in which Ringer´s solution had been administered intravenously to conscious euhydrated healthy adult volunteers of both genders. Here, the value of *k*_10_ is 22 × 10^−3^ min^−1^ (95% confidence interval, CI, 19‐25). Half of the excess fluid in the plasma volume and in the body would then be excreted after a mean of 32 and 100 min, respectively. During the same time, *k*_b_ was 4.7 × 10^−3^ (95% CI, 4.2‐5.1) min^−1^, which is 20% of the urinary excretion.

**FIGURE 2:**
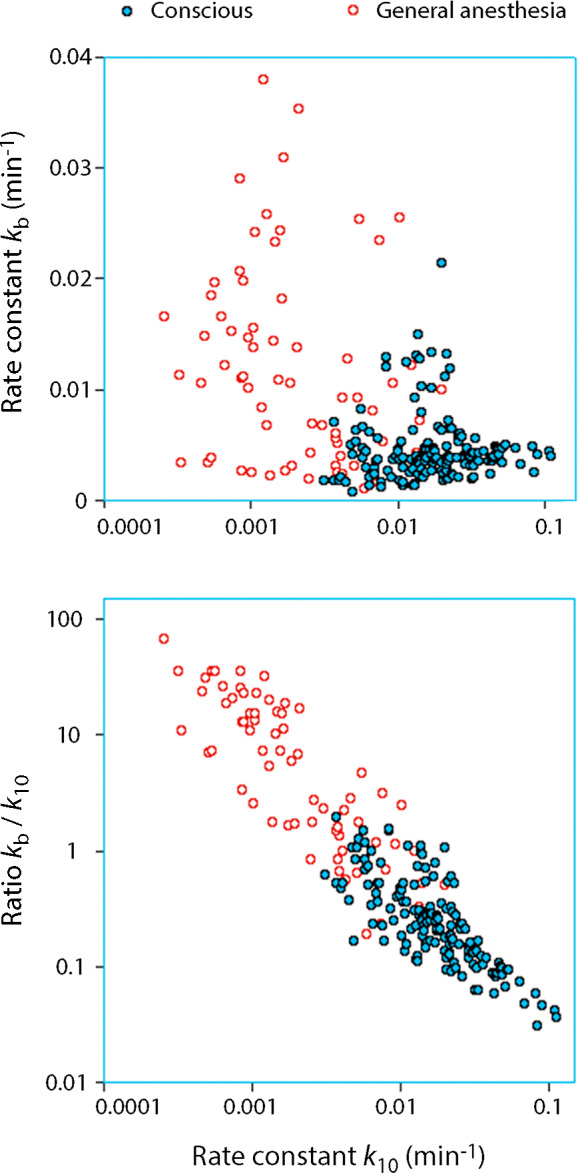
Relationships between *k*_10_ and *k*_b_ in 157 infusion experiments in conscious volunteers and 85 patients receiving crystalloid fluid during general anesthesia. Extensive overlapping. The sum of these two rate constants is the total elimination of fluid volume from the kinetic model and *k*_10_ the fraction accounted for by urine

These parameters change markedly in patients subjected to general anesthesia. Data were obtained on 85 patients from four studies of laparoscopic and open surgery associated with low‐grade blood loss.^23^, ^24^, ^25^, ^26^ Here *k*_10_ was only 3.2 × 10^−3^ min^−1^ (95% CI, 2.4‐3.9) while *k*_b_ increased to 10.1 × 10^−3^ min^−1^ (95% CI, 8.3‐12.0), that is, three times higher than the urine flow rate (Figure 2). Simulations suggest that half of the excess fluid in the plasma volume would then be excreted after 225 min while as much as 80% of the fluid load would remain in the body after 6 hours.

These data show the dramatic inhibition of the diuretic response to volume loading during anesthesia and surgery, which is not new.^11^ However, the data also illustrate the additional problem, namely that the reciprocal increase of *k*_b_ even prevents the kidneys from excreting the entire fluid load as long as it does not equilibrate with the plasma.

**FIGURE 3:**
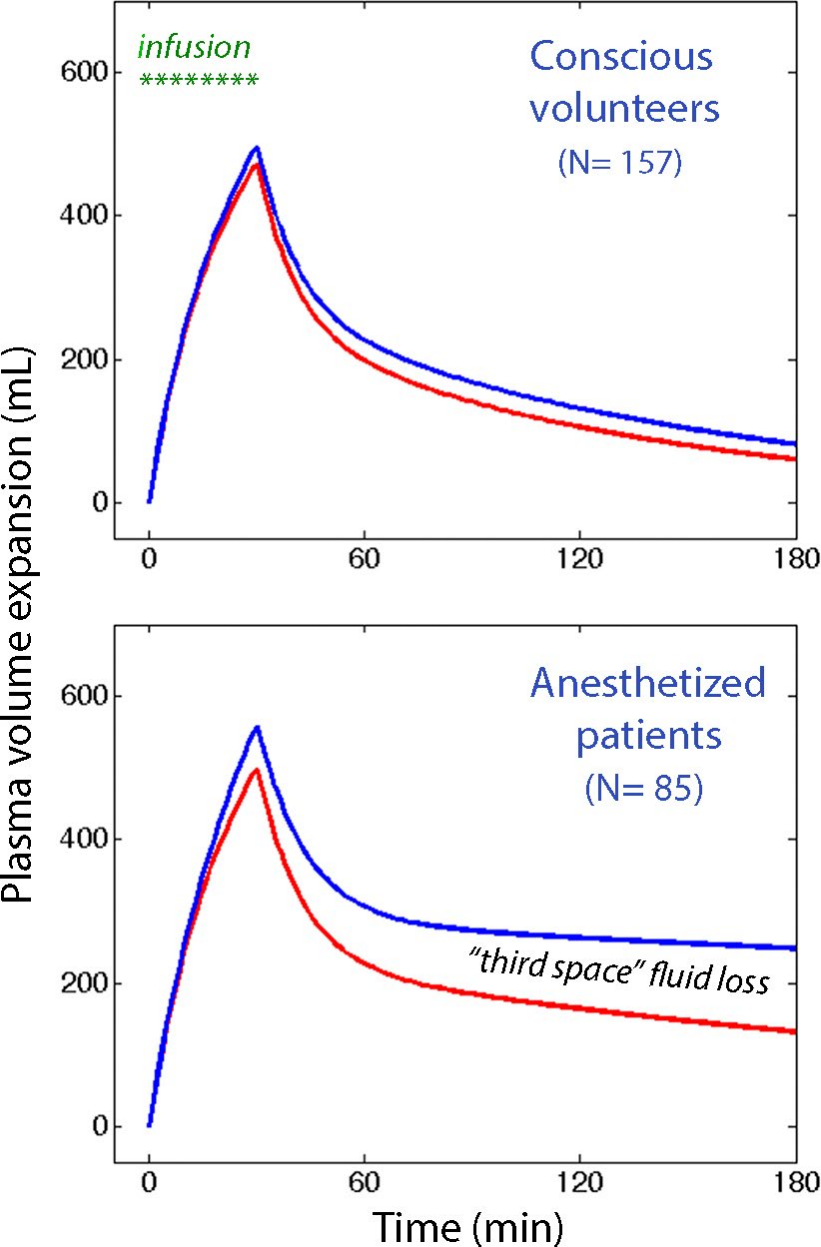
The effect of "thirds‐spacing" of infused fluid in conscious vs anesthetized humans. Simulations based on mean kinetic data from conscious volunteers and anesthetized patients. If elimination of 1 L of Ringers infused over 30 min was determined only by urinary excretion the plasma volume expansion would follow the top curve (blue). In reality, the modeled plasma volume expansion follows the lower curve (red). The difference represents the elimination of fluid that is not in balance with the circulating blood. A similar but larger deficit is present in the interstitial fluid space

The power of "third spacing" to exclude infused fluid from the kinetic system during general anesthesia vs the conscious state is highlighted by the simulation shown in Figure 3. In this worked‐out example, 1 L of buffered Ringer´s is infused over 30 min in a conscious volunteer. Late in the experiment, at 180 min, the plasma volume expansion would be 70 mL, whereas the measured urinary excretion indicates an expansion of 80 mL. If performed during general anesthesia, the same infusion experiment would yield 275 mL and 400 mL, meaning that 125 mL is lost from the plasma, *V*_c_. However, fluid is also missing from the extravascular space, *V*_t_, which is in balance with the plasma. The measured urinary excretion suggests that 573 mL would reside in *V*_t_ at 180 min. If we consider the "third‐spacing" found by kinetic analysis, the true amount of fluid in *V*_t_ would be only 346 mL. The total loss of fluid from the kinetic system is then 125 + (573‐346) = 352 mL. Hence, one‐third of the infused fluid volume no longer equilibrates with the plasma at 180 mL.

## 8 WHAT ARE WE LOOKING AT?

This example illustrates that *k*_b_ should be considered to be a variable that contributes to edema and weight increase after surgery. Another factor that can add to the problem is exudation of protein‐rich fluid due to surgical cutting and inflammation. Very fast infusion of crystalloid promotes edema by disrupting the balance of fluid via *k*_12_/*k*_21_, as already mentioned, but *k*_b_ does not require vigorous volume loading to become important; the inverse relationship with *k*_10_ stands out as being the essential prerequisite.

Freely available articles on the Internet describe the “third space” as part of the interstitium where excessive amounts of fluid accumulate between the cells in cases where the capillary permeability is increased, as occurs in many disease states. By contrast, volume kinetics suggest that some degree of “third‐spacing” occurs even in healthy volunteers. However, importantly, it also suggests that the fraction increases greatly when the urinary excretion is restricted, despite adequate or increased body hydration.

General anesthesia seems to create an ideal situation for “third‐spacing.” Here the diuretic response to fluid loading is strongly inhibited due to the reduction in mean arterial pressure.^11^ The fact that hemorrhage and dehydration without hypotension reduce *k*_b_ sufficiently to make it undetectable^27^ supports the concept that “third‐spacing” is a mechanism aimed at counteracting intravascular volume overload.

What *k*_b_ represents from an anatomical point of view is still unclear. Speculations should consider that the fluid volume, from a kinetic point of view, is not readily exchangeable with the plasma volume during an infusion experiment that may last as long as 3 or 4 hours. Possible sites of fluid accumulation include the gastrointestinal tract, the skin, surgical wounds, the lymphatics, or other special areas of the body. The prominent *k*_b_ in sheep is interesting from the viewpoint that sheep are able to harbor large amounts of fluid in the gut.

No study has yet been performed that aims to limit or prevent fluid losses by *k*_b_. Whether this can be achieved by restrictive fluid therapy or by diuretic therapy during the surgery is not known. However, the possible benefits, if succeeding in this mission, might include hastened postoperative recovery and prevention of late pulmonary edema and other complications associated with fluid overload.^6^

## 9 CONCLUSION

Weight increase is common after surgery and may remain for several days. Poor handling of fluid during the actual surgical procedure, rather than postoperatively, seems to be responsible for the increase in body weight. Population kinetic analysis of fluid volume shifts can detect "third‐spacing" of infused fluid, which means that some of the infused fluid remains in the body but without equilibrating with the plasma. This type of "third‐spacing" is negligible in conscious humans but typically comprises one‐third of the infused fluid volume during surgery.

## 10 COMPETING INTERESTS

RGH holds a research grant from Grifols for studies of 20% albumin.

## Data Availability

The original data used for the calculations can be obtained from the author upon request.
